# Suspension of Mitotic Activity in Dentate Gyrus of the Hibernating Ground Squirrel

**DOI:** 10.1155/2011/867525

**Published:** 2011-05-15

**Authors:** Victor I. Popov, Igor V. Kraev, Dmitri A. Ignat'ev, Michael G. Stewart

**Affiliations:** ^1^Institute of Cell Biophysics, Russian Academy of Sciences, Pushchino, 142290, Russia; ^2^Department of Life Sciences, Faculty of Science, The Open University, Walton Hall, Milton Keynes MK7 6AA, UK

## Abstract

Neurogenesis occurs in the adult mammalian hippocampus, a region of the brain important for learning and memory. Hibernation in Siberian ground squirrels provides a natural model to study mitosis as the rapid fall in body temperature in 24 h (from 35-36°C to +4–6°C) permits accumulation of mitotic cells at different stages of the cell cycle. Histological methods used to study adult neurogenesis are limited largely to fixed tissue, and the mitotic state elucidated depends on the specific phase of mitosis at the time of day. However, using an immunohistochemical study of doublecortin (DCX) and BrdU-labelled neurons, we demonstrate that the dentate gyrus of the ground squirrel hippocampus contains a population of immature cells which appear to possess mitotic activity. Our data suggest that doublecortin-labelled immature cells exist in a mitotic state and may represent a renewable pool for generation of new neurons within the dentate gyrus.

## 1. Introduction

Hibernation is a behavioural and physiological adaptation of endothermic animals which enhances survival during extended seasonal periods of reduced food supply and low ambient temperature when core body temperatures can be close to 0°C. The ability to hibernate is found throughout the class Mammalia and involves differential expression of genes common to all mammals, rather than induction of novel gene products unique to hibernation. Most hibernators periodically interrupt the state of hibernation (torpor) by euthermic episodes or arousal, a process responsible for up to 90% of the energy consumed during hibernation. Previously, using Golgi and electron microscope studies in hippocampal tissue of hibernating ground squirrels we have shown marked structural alterations in the components of neural circuitry, with the reversible retraction of dendritic spines and synapses in CA1 and CA3 [1, 2].

With the exception of hibernators, mammals as a whole are much more sensitive to hypothermia, than are their individual constituent cells. Even though the optimum growth of mammalian cells occurs at 35–37°C, in culture many continue to grow and divide at 25–33°C, although substantially more slowly than at optimal body temperatures. Prolongation of the cell cycle at “suboptimal” (25°C–33°C) temperatures is attributed to a temporal expansion of the G1 and S phases, with G2 being the least sensitive [3]. However, research into the process by which hypothermia affects the cell cycle has been largely neglected. This is despite the fact that work on the response of mammalian cells to lower than optimal temperatures, first published in the mid 20th century, suggested several intriguing effects on cell cycle progression. Although the mechanisms involved remain largely unexplored, a sudden but severe cold shock can be used to generate a high degree of cell cycle synchrony in some mammalian cultures. Other hypothermic regimes can also be used to enrich cultures for G2 cells and to significantly slow otherwise rapid cellular processes, including mitosis [4]. In this respect hibernating mammals provide a rich resource to study the process of cell division and neurogenesis.

Neurogenesis in the mammalians in hippocampus persists through adulthood mainly within the two neurogenic structures, the dentate gyrus of the hippocampus and the subventricular zone of the forebrain [5–8]. In these areas neural progenitor cells continuously divide and give birth to new neurons [5, 6]. Previous studies have demonstrated that behavioural and physiological stimuli, such as learning [9], voluntary wheel running exercise [10], kindling [11], and environmental enrichment [12], enhance hippocampal neurogenesis. However, although the veracity of hippocampal neurogenesis is now generally accepted, there is uncertainty surrounding the extent of adult neurogenesis. Moreover, whether neurogenesis involves only progenitor cells or also more mature hippocampal cells is unclear and the absence of definitive phenotypic markers at ultrastructural level has limited the development of the field. 

Investigations initiated in the 1970s by Kaplan using ^3^H-thymidine light microscopic autoradiography and ultrathin sections at electron microscope level reexamined the initial observations and provided evidence not only that could neurogenesis occur in the adult brain, but also that the cells appear ultrastructurally, similar to sister cells in the dentate gyrus of the hippocampus, one of the structures shown to be neurogenic [12–14]. Also, studies performed by Gould and McEwen demonstrated that mitosis and apoptosis in hippocampus are regulated by adrenal steroids, possibly through excitatory amino acids which may also participate in the development and maintenance of the dentate gyrus [15].

The Siberian ground squirrel *(Spermophilus undulatus) *which has adapted to survive under very low environmental temperatures and a short “summer season” is a unique animal for investigation of phenotypic metabolism changes [16–18]. This is because there are 2 euthermic states: (i) a winter interbout state and (ii) summer animals, with typical features for nonhibernating rodents. In the present study we have utilised this natural hibernation model in the adult arctic ground squirrel to study mitosis at different stages of cell cycle. Our data suggest that immature cells exist in a mitotic state and may represent a renewable pool for generation of new neurons within the dentate gyrus.

## 2. Materials and Methods

### 2.1. Animals

Adult ground squirrels, *Spermophilus undulatus*, of both sexes and 600–700 g in weight, estimated as between 2 and 3 years of age were caught in Yakutiya (Siberia) and kept in individual cages in a cold vivarium under natural photoperiodicity. Food was supplemented with sunflower seeds and carrots, and nesting material was provided *ad libitum*. In November, the animals were individually placed in wooden hibernation boxes (20 × 20 × 25 cm) and transferred to a dark room having a temperature of 1–3°C. Food was not provided during hibernation. Functional states were initially characterized by telemetry of heart rate (HR) and body temperature (Tb), by using a special transducer. Later, continuous monitoring of functional states was performed simply by recording the nest temperature. At the bottom of the wooden animal box, a thermistor (sensitivity 0.2°C) was mounted in the nest bedding. During hibernation, the temperature of the bedding was in the range 1–4°C, whereas in interbout events it increased to 14–20°C. The course of torpor-activity cycles could thus be monitored in individual animals and allowed us to predict the length of a subsequent bout. “Deep torpor” samples were taken from animals killed in the predicted middle of a hibernation bout. In December, hibernation bouts had a 1-week duration, but this became 1.5–2 weeks in January-February. Occasionally, animals had a 3-week bout duration. To measure Tb, the transducer was placed into the rectum at a depth of 5 cm. HR was determined by using electrodes fixed onto the skin of the shoulder blade and the left foreleg. Directly after the start of perfusion procedure, the heart temperature (Th) was determined with an electrical point thermometer (sensitivity 0.2°C).

The functional states were defined as follows: (i) *deep torpor*, in the middle of an extended bout of deep hibernation (usually 3 to 4 days of hibernation bout; brain temperature 2–4°C; HR 6–8 beats per min); (ii) interbout (usually at the second day after natural arousal), *quasiactive animals*, which had increased their brain temperature spontaneously to about 36°C between two hibernation bouts; *S. undulatus *relies on its fat depots and does not need food in these brief intervals of normothermic arousal (HR 150–300 beats per min); (iii) *2.5 h provoked arousal*: 2–2.5 h awakening from the middle of hibernation bout, provoked by transfer to a warm room. In the last case the provoked awakened ground squirrels had a Tb of 34°C (HR 300–400 beats per min).

### 2.2. BrdU Injections

Three animals from each group received an intraperitoneal injection of BrdU (5-bromo-2-deoxyuridine; Sigma) in dose 150 ug/g body weight in sterile 0.9% NaCl solution. The animals were injected according to their group (3-4 animals per group) as follows.

- *Hibernation*. Using nest temperature monitoring (as above) 4 animals were chosen with interbout normothermic interval 2 days. 1 day before entrance in hibernation BrdU injection was done in interbout/normothermic animals (Tb *∼*35-36°C). After 3 days of hibernation the animals were perfused with solution temperature similar to Tb *∼*4–6°C. Tb was measured after decapitation.
- *2.5* *hr provoked arousal*. The injection has been done on animal in deep torpor (Tb *∼*3.5–4°C). After 2.5 hrs of provoked arousal from hibernation the animals were perfused. Tb at the start of perfusion *∼*33-34°C.
- *Normothermic winter animal*. The injection was done 2.5–3 hrs after the provoked arousal. The animal stayed awaked and was perfused 3 days after. Tb at the start of perfusion *∼*36°C.
- *Normothermic summer animal*. Animals were injected 3 days before perfusion. Tb at the start of perfusion *∼*36-37°C.

### 2.3. Tissue Preparation for Immunohistochemistry

Animals were sacrificed with an overdose of ketamine and perfused transcardially with 100 mL physiological saline in 0.01 M phosphate buffer (pH 7.4) (PBS), followed by 300 mL of 0.5% glutaraldehyde and 3% paraformaldehyde in 0.05 M cacodylate buffer (pH 7.4). The brains were stored in the fixative overnight. 50 *μ*m coronal sections were cut on vibrotome (Leica).

### 2.4. Antibodies DCX Immunohistochemistry

All antibodies were diluted in 0.1 M phosphate buffer (pH 7.4) containing 0.05% Triton X-100 and 2% fish gelatin (Sigma) (incubation buffer). The primary antibodies used in this study were monoclonal rat anti-BrdU (cat. no. ab6326, Abcam, UK), 1 : 300, and polyclonal rabbit anti-DCX (cat. no. ab18723, Abcam, UK), 1 : 700. For immunohistochemistry with the peroxidase technique, biotinylated donkey anti-rat IgG (cat. no. 712-065-150, Jackson, USA), 1 : 200 and biotinylated donkey antirabbit IgG (cat. no. 711-065-152, Jackson, USA), 1 : 100, were used as secondary antibodies and detected with avidinbiotin-peroxidase complex (ABC, VECTASTAIN Elite, Vector Laboratories, UK) (9 *μ*L/mL).

### 2.5. BrdU Immunohistochemistry

After quenching excess of aldehyde groups with 1% sodium borohydride in PB for 30 minutes the 50 um sections were incubated in 2 M HCl for 30 min at 37°C and washed in borate for 5 minutes. The sections were blocked in incubation buffer (IB) for 2 hours, followed by incubation in primary antibody in IB overnight at 4°C. After rinses in PB, the sections were incubated in the secondary antibody in IB for 4 hours at room temperature. After another set of rinses, ABC Elite reagent (Vector Laboratories, UK) was applied for 1 hour. As substrate for the peroxidase reaction, diaminobenzidine (DAB, Sigma, USA) was applied for 5 minutes at a concentration of 0.22 mg/mL in Tris buffer (pH 7.4) with 0.01% hydrogen peroxide. Sections were thoroughly washed, mounted, air-dried, dehydrated, and cover-slipped. To control for nonspecific labelling, adjacent sections were incubated without primary or secondary antibody; no labelling was detected following this procedure.

### 2.6. Statistical Analysis

Excel software was used to organize the data. Statistica (StatSoft, Tulsa, OKla, USA) was used to obtain means SDs to perform statistical analyses, and the Kolmogorov-Smirnov and Shapiro-Wilk normality tests to compare distributions (criterion *P* < .05). One-way ANOVA test followed by Bonferroni's or Tukey's unequal N honest significant differences tests was performed with the OriginPro 7.5. All data are presented as a mean ± SD.

## 3. Results and Discussion

**Figure 1:**
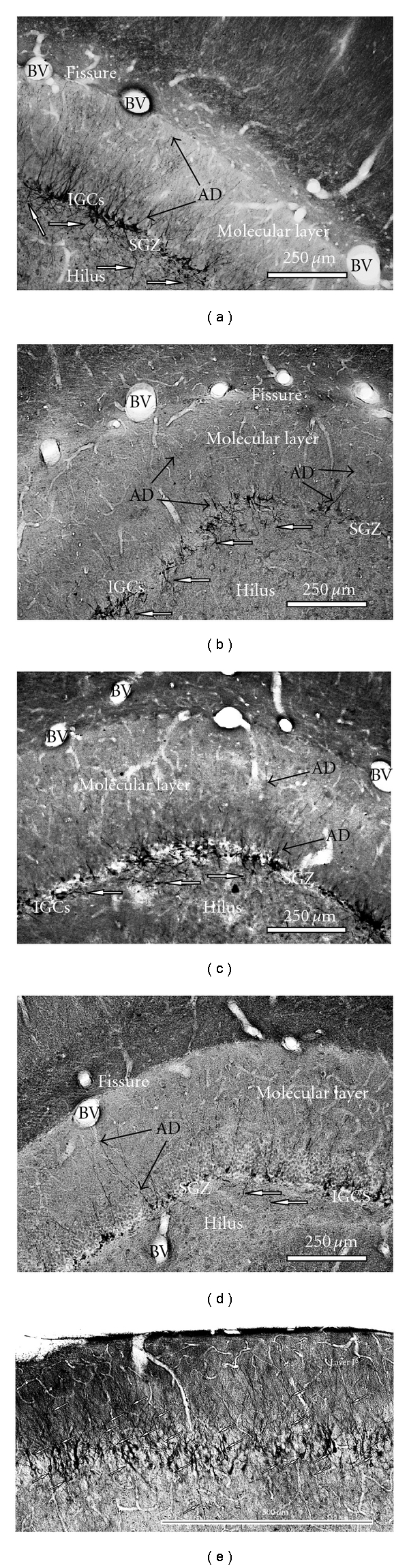
Comparative analysis of DG images of DCX-immunoperoxidase-labelled cells in *S. undulatus* in different functional states: (a) normothermic summer animal; (b) normothermic winter animal; (c) 2.5 h provoked arousal; (d) hibernation. Intensive DCX labelling of immature granule cell dendritic tree was revealed in normothermic summer animals (a) in contrast to winter animals (b–d). Solid arrows show DCX-labelled dendritic trees and their retraction in hibernating state. Open arrows show there is significant retraction of mossy fibres in hibernating *S. undulatus* (d) in comparison to summer animals (a): (e) DCX labelling of neurons in Layer II of entorhinal cortex in summer animal (open arrows). AD: apical dendrite of granule cell; BV: blood vessel; IGCs: immature granule cells; SGZ: subgranular zone of DG.

**Figure 2:**
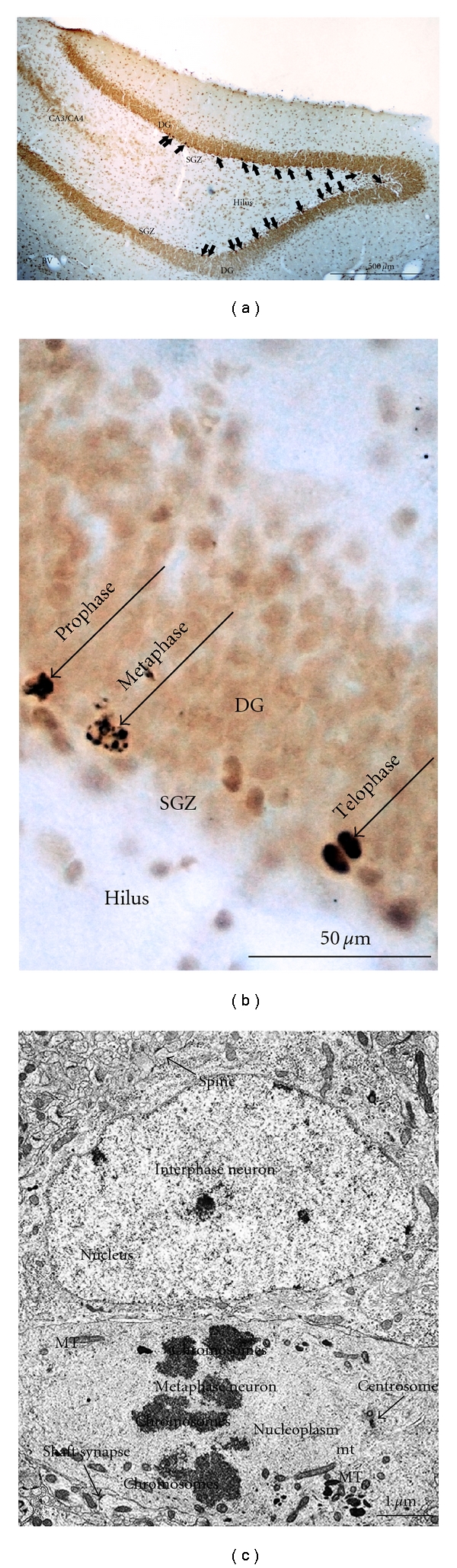
Light and electron microscopic patterns of dividing immature cells in the DG of hibernating animals. Mitotic phases are seldom found in normothermic animals (not shown here). (a and b) are light microscopic analysis of BrdU-immunoperoxidase-positive cells (arrowheads) in the DG of hibernating ground squirrel after injection of BrdU in interbout animal (on 50 *μ*m thick coronal slices). Note the distribution of BrdU-labelled cells in the SGZ. (b) Higher magnification light micrograph demonstrating granule cells where low temperature has halted division showing clearly different mitotic phases: metaphase and telophase due to hypothermia causing entrance into the hibernation state. (c) Ultrastructural details from an electron micrograph (6000× magnification) of an early metaphase immature granule cell from rat hippocampus, (image adapted from a figure shown in Popov et al. [3]), with an interphase neuron located above the metaphase cell. Both immature interphase and metaphase granule neurons have synapses (interphase neuron-dendritic spine and metaphase one-shaft synapse) which are absent in mature granule cells of hippocampus of either ground squirrel or rat. BV: blood vessel; DG: dentate gyrus; SGZ: subgranular zone; mt: microtubules; and MT: mitochondria.

**Figure 3:**
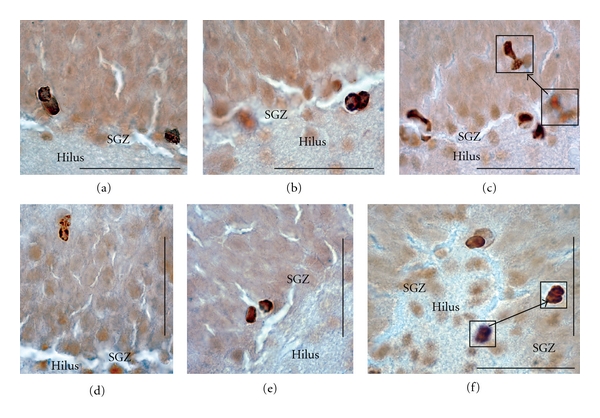
Light microscopic immunohistochemical demonstration of the BrdU-labelled doublets in DG as a result of apparent mitotic division of cells. Postmitotic BrdU immunoperoxidase-labelled cells in different zones of DG on coronal slices (50 *μ*m thick). (a–c) Localization of postmitotic cells near subgranular zone (SGZ). (d–f) Possible migration of postmitotic cells from SGZ (e) to supragranular zone of DG (d). (f) Because of the thickness of brain slices different BrdU-labelled cells are frequently located at various depths in the 50 *μ*m slice, in (f) (inset) the same cells can be seen at different focal planes. Scale bar = 50 *μ*m.

Figures 1(a), 1(b), 1(c) and 1(d) show the distribution of immature neurons with DCX-immunoperoxidase staining in the dorsal portion of dentate gyrus in the 4 different functional states as previously described in [19], which corresponds to the distribution of newborn neurons in this brain region observed using other markers for newly generated neurons [19–23]. No labelling of other pyramidal neurons or interneurons was seen in hippocampus. The SGZ appears discontinuous because DCX-labelled granule cells formed clusters [3, 19] and the appearance of the labelled cells was similar to mature granule cells. DCX-positive cells were mainly found in the SGZ at the hilar border. Labelled cells with branched apical dendrites were found only in summer animals, and many had bifurcating and trifurcating processes (Figure 1(a)). Clusters of DCX-labelled neurons consisted of 4–6 neighbours. Detailed analysis of such images has been made previously [3, 19]. Comparative analysis of DCX-labelled dentate gyrus (DG) cells in summer (Figure 1(a)), and hibernating animals (Figure 1(d)), shows retraction of dendritic branches during hibernation supporting previous data on reversible retraction of dendritic branches [1]. The appearance of these DCX-labelled neurons was similar to that of DG cells. It is notable that in summer, normothermic animals showed intensive labelling of neurons in Layer II of entorhinal cortex (Figure 1(e)). Axons of neurons from Layer II of the perforant path input directly to the DG and both entorhinal cortex and DG participate in spatial memory [24]. DCX-immunoreactive cells occur in the piriform cortex in adult mice and rats [25]. These data suggest that immature neurons may persist into adulthood rodents and that these cells appear to undergo development and differentiation. It is possible that these DCX immunoreactive neurons enable the high degree of plasticity of the neuronal network. Our study was designed only to show that mitotically active granule cells were localized in the DG. Theoretically BrdU insertion occurs during DNA repair and may not detect a distinct proliferative state. However; we did not observe BrdU labelling of either pyramidal neurons or interneurons in different brain regions where there could be intensive DNA repair. Hypothermia can stop the cell in any phase of the cell cycle, though mainly the G2 phase. For mitotically active intestinal epithelial cells during hibernation the mitotic index in the intestinal crypts of ground squirrels is *∼*2%, and then it climbs to *∼*20% two hours after arousal [26]. Here we have studied torpid animals after BrdU injection in interbout animals. Entrance into hibernation stimulated suspension of the cells in different phases of the cell cycle. Figure 2 shows BrdU immunoreactive cells in 3 different phases of mitosis: metaphase, anaphase, and telophase including ultrastructural patterns of metaphase immature granule neuron. Figure 3 shows that after telophase many BrdU-labelled cells in the DG formed cellular doublets. BrdU-labelled cells such as those shown in Figures 2 and 3 originate apparently from the DG layer supporting the idea [3] that mitosis occurs within DCX clusters.

Previously using serial ultrathin sections we found [3] that, within DCX-labelled clusters of immature granule cells, neurons are present during mitosis. Popov et al. [3] show interphase and metaphase granule cells where definitive markers are somatic synapses that are absent in mature granule cells. Uncovering such an event is exceptional because the cell cycle time is approximately 24.7 hours [25, 27] whilst the duration of mitosis has been assessed as only 10% of total time of the cell cycle. By determining the length of the cell cycle for dividing cells and the total number of dividing cells [27], it may be estimated that approximately 9,000 new cells are generated in the adult rat dentate gyrus each day. The daily regulation of the hippocampal neurons, an apparent daily change in the number of S-phase cells, has been reported in the hilus [28]. Thus, proliferation in the hilus seems to depend on the time of the day.

Cell divisions in other mammalian tissues (e.g., tongue epithelium, intestinal epithelium, and skin) are associated with specific periods in the day [29–31]. In the hippocampus [32], it has been observed that M-phase cells show a clear day/night variation, with a significant increase during the night but the number of S-phase progenitors remains unchanged across the day [32]. It is therefore very difficult to demonstrate mitotic cells in dentate gyrus of adult rodents amongst more than 2 millions [33] granule cells. However, in this connection hibernation of Arctic ground squirrel presents unique model for accumulation and the study of different stages of cell cycle including mitosis.

## 4. Conclusion

Our immunohistochemical studies of doublecortin- (DCX) and BrdU-labelled cells in the dentate gyrus of the adult arctic ground squirrel hippocampus have demonstrated a population of immature cells which appear to exist in a mitotic state and may potentially represent a renewable pool for generation of new neurons within the dentate gyrus.
